# Fabrication and Structural Design of Micro Pressure Sensors for Tire Pressure Measurement Systems (TPMS)

**DOI:** 10.3390/s90301382

**Published:** 2009-02-27

**Authors:** Bian Tian, Yulong Zhao, Zhuangde Jiang, Ling Zhang, Nansheng Liao, Yuanhao Liu, Chao Meng

**Affiliations:** State Key Laboratory for Mechanical Manufacturing System, Institute of Precision Engineering, Xi’an Jiaotong University, Xi’an 710049, P.R. China

**Keywords:** Piezoresistive, TPMS, Pressure sensor, FEM

## Abstract

In this paper we describe the design and testing of a micro piezoresistive pressure sensor for a Tire Pressure Measurement System (TPMS) which has the advantages of a minimized structure, high sensitivity, linearity and accuracy. Through analysis of the stress distribution of the diaphragm using the ANSYS software, a model of the structure was established. The fabrication on a single silicon substrate utilizes the technologies of anisotropic chemical etching and packaging through glass anodic bonding. The performance of this type of piezoresistive sensor, including size, sensitivity, and long-term stability, were investigated. The results indicate that the accuracy is 0.5% FS, therefore this design meets the requirements for a TPMS, and not only has a smaller size and simplicity of preparation, but also has high sensitivity and accuracy.

## 1. Introduction

Nowadays, there is growing interest in Tire Pressure Measurement Systems (TPMS), which has led to the development of sundry pressure sensors. Despite the abundant evidence that tires are definitely one of the most vital safety components on a vehicle, but a tire can lose up to half of its air pressure without appearing to be underinflated and most people ignore the state of their tires so data shows that nearly 250,000 accidents per year occur in the United States alone due to low tire pressure. The place where the rubber meets the road affects traction, handling, steering, stability and braking [1]. Because of this, a sudden tire failure can induce serious consequences, more so if it occurs when driving at highway speeds. To avoid the above, the concept of a TPMS was introduced. Direct TPMS contain small sensors for pressure and acceleration that are installed inside the tires. These sensors monitor tire pressure and continually feedback this information, via wireless signals, to the vehicle’s electronic control unit (ECU). If the tire pressure falls below required limits, a warning signal is sent to the instrument panel that alerts the driver about the problem. Since the discovery of the piezoresistive effect, piezoresistive sensors have been widely employed in mechanical signal sensing, which plays a very important role in TPMS. The piezoresistive sensor has the advantages of simple fabrication and stable performance compared with the capacitive sensor, which has a complicated fabrication process and signal circuits, and moreover, the performance of this last type of sensor is easily affected by circumstantial impurities. The SOI wafer is another choice for the fabrication of pressure sensors, but the expensive cost of SOI materials and the complicated process for implanting the resistance and down-lead have restricted its applications in autos. The piezoresistive pressure sensor is formed by diffusing impurities onto a semiconductor and has the advantages such as easy fabrication, superior linearity and high sensitivity [4]. The measurement range of a TPMS should be 0 to 1 MPa, and it is mounted and used in the tire under circumstances where it may experience changes of temperature from about 0 to 85°C. By applying a simplified processing circuit in the piezoresistive pressure sensor, a correction and compensation circuit is usually added to overcome the nonlinearity caused by any poor temperature characteristics thereof.

Merits of the Micro Electro Mechanical systems (MEMS) technology are proven in the manufacture of sensing elements of small and definite size. The desired measurement range, bandwidth, and sensitivity can be easily achieved by adjusting the size of the sensing elements. In previous work, a structural model of a sensor is built by the finite element method (FEM) using the ANSYS software to calculate the structure stress and to define the size of the sensor. The FEM is widely adopted for stress analysis, thermal effect reduction, packaging design and reliability of enhancements to piezoresistive sensors [5]. In a structural simulation, the FEM is very effective in producing the visualized stiffness, the strength and also in the mathematical minimization of the weight, the materials and the costs. FEM expresses the visualization details of where the structures deform or twist, and indicates the distribution of stresses and the displacements. FEM software possesses an abundance of simulation options for controlling the complex system of both modeling and analysis [6]. In the development of this pressure sensor, bulk micromachining was applied to the fabrication of the sensor. This method is cheap and easily realized. For the bulk micromachining technique [7], a KOH solution is usually used to etch the silicon substrate to form cavities in a trapezoid shape with a 54.7° inclined wall, which allows for diaphragm deformation. Since the silicon substrate is relatively thick and the bottom region etched is much larger than the top region where the pressure diaphragm defines. In addition, investigation shows that decreasing the thickness of diaphragm and widening the bottom region can give a smaller size to satisfy the measurement requirement of sensors [8]. We have used a 4 inch silicon wafer and a Pyrex7740# glass wafer for silicon-glass anodic bonding, applying a silicon-glass anodic bonding technique instead of Si-Si bonding which is operated in 1000°C due to the destruction of the Al wire [11]. Ti is placed between the Al and the resistance for decreasing the contact resistance. The packaging adhesive is stuffed with impurities for lowing residual stress. In the end, the sensor characteristics were listed its and sensitivity, range, and linearization were also analyzed in this paper.

## 2. Structural designs

### 2.1. Detection principle

For a membrane type piezoresistance pressure sensor, the stress state on the resistors can be assumed to be in a plane stress (*σ_z_* = *σ_xz_* = *σ_yz_* = 0) condition and there are the shear stress *σ_xy_* and the normal stress *σ_x_*, *σ_y_*. Two uniaxial stresses are defined: a longitudinal stress, *σ_l_* (the uniaxial stress, electric field and current of this piezoresistor are all in the same direction) and a transverse stress, *σ_t_* (the uniaxial stress is in perpendicular to the direction of electric field and current) for the membrane type piezoresistance sensor application[12]. The relationship between resistivity variations and stress change can be expressed as follows (Eq. (1)):

(1) $$\frac{\Delta \rho }{\rho }=\pi_{l}\;\sigma_{l}+\pi_{t}\;\sigma_{t}$$

where

(2) $$\pi_{l}=\pi_{11}+2(\pi_{44}+\pi_{12}-\pi_{11})(l_{1}^{2}\;m_{1}^{2}+l_{1}^{2}\;n_{1}^{2}+m_{1}^{2}\;n_{1}^{2})$$

(3) $$\pi_{t}=\pi_{12}-(\pi_{44}+\pi_{12}-\pi_{11})(l_{1}^{2}\;l_{2}^{2}+m_{1}^{2}\;m_{2}^{2}+n_{1}^{2}\;n_{2}^{2})$$

In Eq. (2) and Eq. (3) *π_l_* is the longitudinal piezoresistance coefficient and where *π_t_* is the transverse piezoresistance coefficient. *π_ij_* is the piezoresistance coefficients defined as a 6 × 6 matrix. In order to get high sensitivity, the four piezoresistors on the membrane which form Wheatstone bridge should be located on the edge of square chip. It is well known that there is higher piezoresistive sensitivity along the single crystal direction [110] and [**11̄0**] for the (100) p-type silicon. Then the four resistances (*R*_1_, *R*_2_, *R*_3_ and *R*_4_) of the Wheatstone bridge are located on the edge of the chip along the crystal direction [110]. The value of longitudinal piezoresistive coefficient to the resistances *R*_1_ and *R*_3_ is expressed as $\pi_{l}=\frac{1}{2}\pi_{44}$. The value of transverse piezoresistive coefficient to the resistances *R*_1_ and *R*_3_ is expressed as $\pi_{t}=-\frac{1}{2}\pi_{44}$. However the value of longitudinal piezoresistive coefficient to the resistances *R*_2_ and *R*_4_ is expressed as $\pi_{l}=-\frac{1}{2}\pi_{44}$, the value of transverse piezoresistive coefficient to the resistances *R*_2_ and *R*_4_ is expressed as $\pi_{t}=\frac{1}{2}\pi_{44}$. Here, *π*_44_ is piezoresistive coefficient of shear direction. Therefore, under an applied pressure, the resistance change that leads to the unbalance of Wheatstone bridge can be directly converted into a voltage signal. For Δ*R*<<*R*, Eq. (4) indicates the relationship between voltage and resistance:

(4) $$\Delta V=\frac{r}{{\left(1+r\right)}^{2}}\;(\frac{\Delta R_{1}}{R_{1}}-\frac{\Delta R_{2}}{R_{2}}+\frac{\Delta R_{3}}{R_{3}}-\frac{\Delta R_{4}}{R_{4}})V_{\mathrm{in}}$$

where r=R2R1=R3R4, Δ*R_i_* is the change of ith resistance, *R_i_* is the ith zero-stress resistance, *V_in_* is bridge-input voltage, and Δ*V* is the differential output voltage.

For *R* = *R*_1_ = *R*_2_ = *R*_3_ = *R*_4_ in Eq. (4), the mechanical stress, the comprehensive change of resistances (Δ*R*) and the output voltage relation can be expressed as follows, which neglects the dimensional change:

(5) $$\frac{\Delta V}{V_{\mathrm{in}}}=\frac{\Delta R}{R}=\frac{\Delta \rho }{\rho }=\pi_{l}\;\sigma_{l}+\pi_{t}\;\sigma_{t}$$

The mechanical stresses obtained by FEM should be transformed into output voltage in such a way that the simulation stress value can be applied to predict the equivalent output electrical signal. Eq. (5) indicates the relationship among output voltage, resistance and stress variation.

### 2.2. FEM analysis

In order to study the performance of the pressure sensor, the FEM (finite element method) was adopted to simulate the mechanical behaviors of the pressure sensor, such as displacement [14], pressure load and so on. As a design method, the FEM establishes a complete model which could be analyzed, adjusted, and optimized before the design is accomplished. This kind of powerful simulation tool has great advantages in improving both the standard of engineering designs and the technique of the design process in many industrial applications and institutional research. As well as the introduction of FEM, it is primarily through improved initial prototype designs using FEM that testing and development have been accelerated [15].

Before fabrication, the size of the diaphragm used in the pressure sensor should be determined by proper analysis and design. The size of the diaphragm is actually determined by the desired measurement range of the sensor, maximum strain and sensitivity. The diaphragm deformation can be simulated as a square shell or plate with four edges clamped under a uniform normal pressure that can be derived as follows.

The maximal stress occurs on the middle of the four sides is given by Eq. (6):

(6) $$\sigma_{\text{max}}=\frac{0.308\;{\mathrm{pa}}^{2}}{h^{2}}$$

where the pressure is represented by *p*, the length of diaphragm is represented by *a*, and the thickness of the diaphragm is represented by *h*.

The maximal strain has relationships with stress shown in Eq. (7):

(7) $$\varepsilon =\frac{1-\nu }{E}\sigma$$

where the Young’s modulus E is 130 Gpa, and the Poisson ratio *ν* is 0.18. For the requirements of TPMS, the scale of pressure is 0 to 1Mpa, and strain range should be from 400 to 500 micro strains. In the range of strain, the sensor could achieve high linearization and sensitivity, which structural parameters can be determined by Table 1:

**Table 1.** Sensor parameters.

	**Length (***μm***)**	**Width (***μm***)**	**Thickness (***μm***)**
**Silicon chip**	1,500	1,500	400
**Silicon membrane**	520	520	30

**Figure 1.**
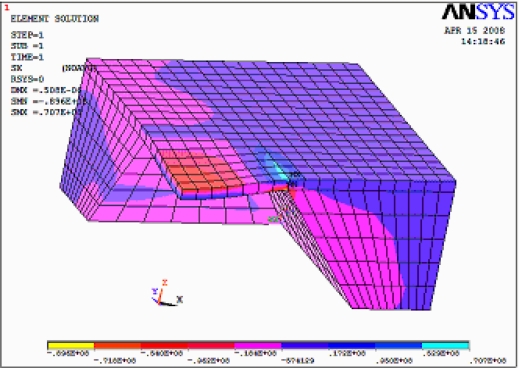
X-direction stress distribution, the gauge of most strain is 707 micro strains that concentrated in the blue area.

The quarter finite element models of the pressure sensors were established since a pressure sensor device with C type structure for a solid model has a quartered symmetry. The x-direction stress distribution is shown in Figure 1. The zone of stress concentration is located at the edge of the membrane, where the resistors are implanted.

**Figure 2.**
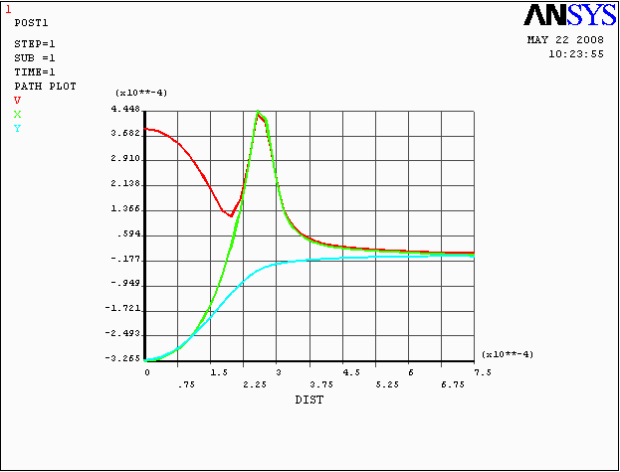
X-direction strain distribution curve, V, X and Y represent the result of von mises, transverse, and longitudinal respectively.

Figure 2 shows the stress distribution along the path from the center to the edge of model and illustrates the relationship between path and stress. In Figure 2, it could be observed that the stress decreased as the increase of displacement from the center of diaphragm diverge. The stress concentration zone where the resistors were implanted was located at the edge of the membrane.

For a diaphragm size of 520 μm by 520 μm with a 30 μm diaphragm thickness, comparison between the theoretical formula of the diaphragm and the ANSYS result shows that the simulation result agreed very well with the approximate solutions of Westergaard and Guckel, respectively [16]. Accordingly, we may conclude that ANSYS is an efficient method for calculation of the design for the given size of a diaphragm.

## 3. Sensor fabrication

In this work, surface and bulk MEMS techniques are used to fabricate the sensing elements. Wet or dry etches are available for bulk micromachining. Usually, silicon wafers are used as substrates which can be anisotropically wet etched for bulk micromachining to form highly regular structures [17]. This etch has the advantage of the fact that silicon has a characteristic crystal structure, which means its atoms are all arranged periodically in lines and planes. Certain subtle planes have weaker bonds and are more easily to etch. The etch results in pits that have inclined walls, with the incline angle being a function of the crystal orientation of the substrate. This type of etching is inexpensive and was generally used in early, low-budget research. Typically, wet etching uses alkaline liquid solutions, such as potassium hydroxide (KOH) or tetramethylammonium hydroxide (TMAH) to dissolve the exposed silicon which remains after the photolithography masking step. These alkaline solutions dissolve the silicon in a highly anisotropic way, with some crystallographic orientations dissolving up to 1,000 times faster than others. Such an approach is often used with very specific crystallographic orientations in the raw silicon to produce V-shaped cavities with definite angles. The surface of these cavities can be etched atomically smooth if the process is carried out correctly, and the dimensions and angles can be precisely defined.

**Figure 3.**
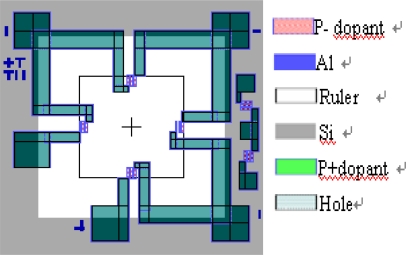
Schematic of a PZT pressure sensor.

We used a 4 inch n-silicon wafer and a Pyrex 7740#glass wafer to bond silicon and glass [18]. The thickness of silicon wafer was 400 μm and its orientation was <100>. The fabrication and the packaging processes were comprised of several steps; and a schematic view of the device layout was shown in Figure 3.

The Si wafer was cleaned and oxidized, and then both sides of the Si wafer were patterned by lithography technologies. The shapes of the resistances were formed on the pressure membrane side with the Wheatstone bridge configuration, which was doped in p-Si. The ion implantation of dosage and the energy is necessary for the goal value of piezoresistance. The density is at the dose of 2.0× 10^15^ cm^−2^ with the energy of 80 keV and the value of piezoresistance is about 25 Ω per square of the piezoresistance width (25 Ω/square). Then the surface of the silicon wafer was deposited with silicon nitride (Si_3_N_4_), which was based on the low pressure chemical vapor deposition (LPCVD) process [19].The Si_3_N_4_ layer is about 0.1 μm thick to protect the Wheatstone bridge circuit of the gauge silicon layer. LPCVD was induced to grow the protecting layer which based on the equations as follows:

(8) $$\begin{array}{l} {\mathrm{SiH}}_{4}\to \mathrm{Si}+{2H}_{2}↑\\ {3\mathrm{SiH}}_{2}{\mathrm{Cl}}_{2}+{4\mathrm{NH}}_{3}\to {\mathrm{Si}}_{3}N_{4}+6\mathrm{HCl}+{6H}_{2}↑ \end{array}$$

The cavity area was formed on the other side with a square section diaphragm (520 μm × 520 μm) which produced the silicon film of the appointed thickness. Then a potassium hydroxide (KOH) anisotropic etching with a density of 25% was utilized to obtain a silicon diaphragm whose thickness was monitored with a surface profiler instrument. The size of the quadrate window designed was 1,044 μm × 1,044 μm. A magnetic stirrer was used to mix the KOH solution to keep the thickness of the silicon uniform.

For the purpose of firmly connecting Al wire with P-Si resistance, and reduction of contact resistance, there should be z heavily doped p+Si between the wire and lightly doped P-Si resistance. The contact resistance can be assumed to obey the equation:

(9) $$R_{c}=\frac{\rho_{c}}{L\times Z}$$

The contact resistance *R_c_* is determined by the contact resistance coefficient *ρ_c_* and the contact area with a length of *L* and a width of *Z*. It is effective to adopt low *R_c_* by using the conduct with low *ρ_c_* when the contact area is determined.

Aluminium is the most commonly used metal for forming electrodes in the fabrication of MEMS and in the IC industry due to its low cost, high conductivity, good stability at room temperature, etc. Moreover, it is especially suitable for forming ohmic contacts as it is a good oxide absorber during thermal annealing of Al/Si contacts. However, Al spikes form through the interface layers and into the silicon substrate due to higher temperature of about 300°C. This reaction is due to the high contact resistance and creepage. Instead of the conventional process of Al wire down-lead, which was directly connected with the silicon layer by using the Al-Si alloy, a structure of double deck Al-Ti was induced using pressure vapor deposition (PVD). The deposition of Ti (100 nm) could protect the Al from reacting with Si, furthermore, Ti adheres well with Al and Si. The value of the contact resistance between silicon with different metal scan be inferred from the Table below:

**Table 2.** Contact resistance between silicon and metal.

**Type**	**Contact resistance/Ω**					
	Al	PtSi	Mo	Ni	Cr	Ti
n-Si	0.09	0.02	0.08	0.02	0.03	0.01
p-Si	0.03	0.02	0.06	0.02	0.04	0.01

**Figure 4.**
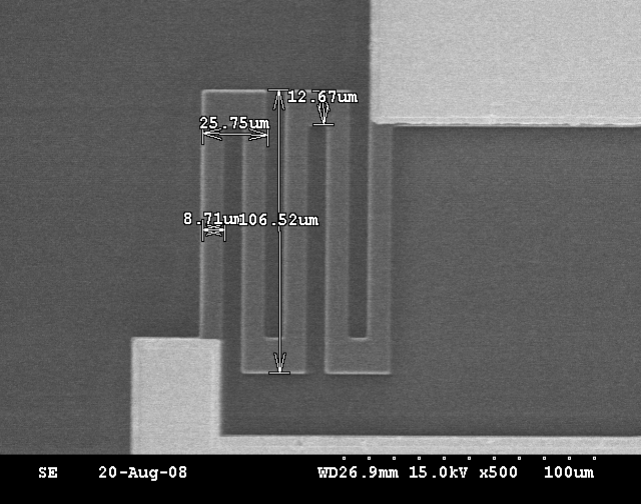
SEM image of resistances, the size of resistance respectively is 106.52 *μm* length and 8.71 *μm* width.

It could be easily seen that the contact resistance between silicon and Ti is lower than others. The fabricated resistances are shown in Figure 4.

The fabricated wafer sputtered with Al wire was anodically bonded onto the Pyrex 7740# glass wafer. Residual stress will be induced when the bonding temperature is above 300°C, although the higher bonding temperature will improve the bonding strength. This result is caused by the changes of thermal coefficients which are induced from the glass and silicon. On the contrary, lower bonding temperatures will decrease the bonding strength. In this study, we operated the bonding process under temperatures of about 200°C–250°C and a voltage about 500 V. Under these circumstances, the bonding strength will be kept high, and the operations under low temperature avoid higher residual stress *effect*.

## 4. Packaging

**Figure 5.**
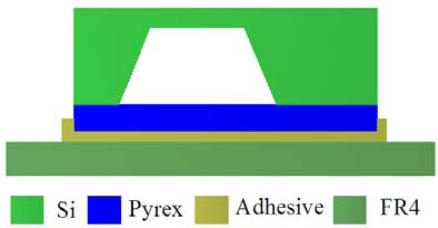
Schematic of packaged sensor.

The packaged sensor is comprised of a silicon chip, adhesive layer and printed circuit board (PCB) substrate. The sensor is attached to the PCB by a resin adhesive which has good electric, thermal and mechanical performance. The schematic of the packaged sensor is shown in Figure 5. Being a part of TPMS, the pressure sensor is mounted in the tire which keeps the temperature of operation between 0 to 85°C.The residual stress of packaging material is determined by the thermal expansion coefficients (CTE) and Young’s modulus [20]. Different materials will cause package-induced thermal stress as the temperature changes and lead to output voltage variations of the sensor. The packaging residual stress will affect the output of the sensor signal, which should be considered in the structural design of the pressure sensor. For the reasons above, the material properties of the adhesive layer should be considered in this study.

The packaging resin is a kind of macromolecular compound material which is comprised of epoxy resin, activator, stuffing and additive(s). The content of oxidized silicon stuffing affects the properties of the packaging resin, the more content stuffed, the more value of CTE decreased, whereas, excessive stuffing will cause the resin be more viscoelastic, which is harmful to the molding process. Hence, the resin is stuffed with the spherical oxidized silicon powder instead of a conventional crystalline one. The spherical one has the characteristics of lower thermal conductivity and lower CTE than the crystalline one.

## 5. Results and analysis

**Figure 6.**
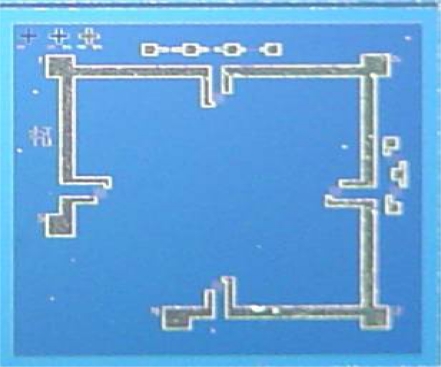
The fabricated sensor.

**Figure 7.**
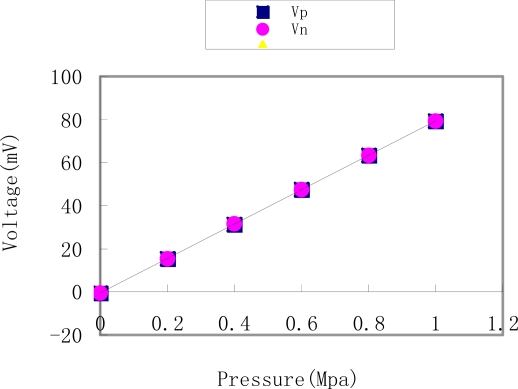
Output of the sensor.

The schematic of the fabricated pressure sensor was shown in Figure 6. About 88% oxidized silicon is stuffed in the resin adhesive to decrease the CTE of the packaging resin to almost 10 × 10^−6^/°C, similarly, 3% silicon oil is added to decrease the Young’s modulus, which causes residual stress at low levels. The output voltage as a function of the applied pressure sensor, which implies the sensitivity and linearity, is listed and illustrated in Table 3 and Figure 7. This experiment done under 1.5 V DC showed the output of the pressure sensor was in the range of 0-1 MPa. The pressure sensor with a nonlinearity of the differential pressure of less than 10% output. The static sensitivity showed the sensor’s ability to detect pressure changes and was a critical parameter for the pressure sensor. According to Table 3, it was known that the sensitivity of the pressure sensor was 79.76 μV/kPa, the linearity was 0.5%, repeatability was 0.04% and accuracy was 0.55%.

## 6. Conclusions

Through use of the technologies of anisotropic chemical etching and glass anodic bonding, a TPMS pressure sensor based on the piezoresistive principle was demonstrated and manufactured. Simplified micromachining techniques were applied to fabricate the pressure sensor. A double decked Al-Ti down-lead structure was used to decrease the contact resistance and obtain stable performance at high temperatures. FEM was applied to analyze the detection principle of the proposed sensor. The effects of thermal residual stress were considered and decreased by stuffing the packaging resin. Results showed that the desired pressure range and sensitivity could be easily obtained by adjusting the size of the sensing element. The sensitivity and measurement range of this pressure sensor reached to 79.76 μV/kPa (voltage output pressure) and from 0 to 1.0 MPa, respectively. The linearity, accuracy and the repeatability indicated that the reliability of the sensors for detecting pressure in tires, moreover, the linear output simplified the design of signal circuits more than capacitive sensora which need more expensve circuits and large sizes after packaging. Comprehensive results suggested that the pressure sensor for TPMS was a better choice for measuring pressure.

## Figures and Tables

**Table 3.** Output of the sensor (Vp is process of pressure growing, and Vn is the decreasing process).

**Pressure(MPa)**	**0**	**0.2**	**0.4**	**0.6**	**0.8**	**1**

**Vp(mV)**	−0.47	15.50	31.41	47.44	63.35	79.29
**Vn(mV)**	−0.48	15.53	31.46	47.49	63.42	79.29

## References

[1] (2004). Schatz, 0. Recent trends in automotive sensors. IEEE Proc. Sensors.

[2] Eddy D. S, Sparks D. R. (1998). Application of MEMS technology in automotive sensors and actuators. Proceedings of the IEEE.

[3] Fleming W.J. (2001). Overview of automotive sensors. IEEE Sensors J.

[4] Kanda Y. (1991). Piezoresistance Effect of Silicon. Sens. Actuat.

[5] Pancewicz T., Jachowicz R., Gniazdowski Z., Azgin Z., Kowalski P. (1999). The Empirical Verification of the FEM Model of Semiconductor Pressure Sensor. Sens. Actuat.

[6] Kronderfer R.H., Lommansson T.C. (2002). Direct calculation of sensor performance in a FEA model. IEEE Proc. Sensors.

[7] Hsu Tai-Ran (2002). MEMS & Microsystems Design and Manufacture.

[8] Gregory T.A. (2003). Micromachined transducers soucebook.

[9] Jiang Z D, Zhao Y L. (2001). Research and characteristic measurement of micro silicon pressure sensor. J. Funct. Mater. Device.

[10] Bao M. (2005). Principles of MEMS Devices.

[11] Zhu Z.H., Ejeckam F.E., Qian Y., Zhang J., Zhang Z., Christenson G. L., Lo Y. H. (1997). Wafer bonding technology and its applications in optoelectronic devices and materials. IEEE Sel. Top. Quantum Electron.

[12] Zhong Z.W. (2002). Calibration of a piezoresistive stress sensor in (100) silicon test chips. Proc. Elect. Packag. Tech Conf.

[13] Lee C.-C., Peng C.T., Chiang K.-N. (2006). Packaging effect investigation of CMOS compatible pressure sensor using flip chip and flex circuit board technologies. Sens. Actuat.

[14] Peng C.T., Lin J.C., Lin C.T. (2004). Analysis and validation of thermal and packaging effects of a piezoresistive pressure sensor. J. Chin. Inst. Eng.

[15] Babuska I., Banerjee U., Osborn J.E. (2004). Generalized Finite Element Methods; Main Ideas, Results, and Perspective. Int. J. Computat. Meth.

[16] Ko H.S. (2007). Novel fabrication of a pressure sensor with polymer material and evaluation of its performance. J. Micromech. Microeng.

[17] Fuller L. F, Sudirgo S. (2003). Bulk micromachined pressure sensor. UNIVERSITY GOVERNMENT INDUSTRY MICROELECTRONICS SYMPOSIUM.

[18] Guan R.F. Die bonding process research for SOI membrane pressure sensor.

[19] Spitzlspreger G. (2003). Very brief introduction to ion implantation for semiconductor manufacturing.

[20] Peng C.T., Lin J.C., Lin C.T., Chiang K.N. (2005). Performance and package effect of a novel piezoresistive pressure sensor fabricated by front-side etching technology. Sens. Actuat. A.

